# “Always look at the outliers”: in memoriam Leif Bertilsson

**DOI:** 10.1007/s00228-024-03667-9

**Published:** 2024-04-03

**Authors:** Julia Stingl, Eleni Aklillu, Collen Masimirembwa, Teh Lay Kek

**Affiliations:** 1 Institute of Clinical Pharmacology, University Hospital RWTH Aachen, Wendlingweg 2, 52074 Aachen, Germany; 2 Global Health Pharmacology and Therapeutics, Department of Global Public Health, Karolinska Institutet, Stockholm, Sweden; 3 African Institute of Biomedical Science and Technology, Wilkins Hospital Harare, Harare, Zimbabwe; 4 Faculty of Pharmacy, UiTM Puncak Alam Campus, Bandar Puncak Alam, Selangor, Malaysia


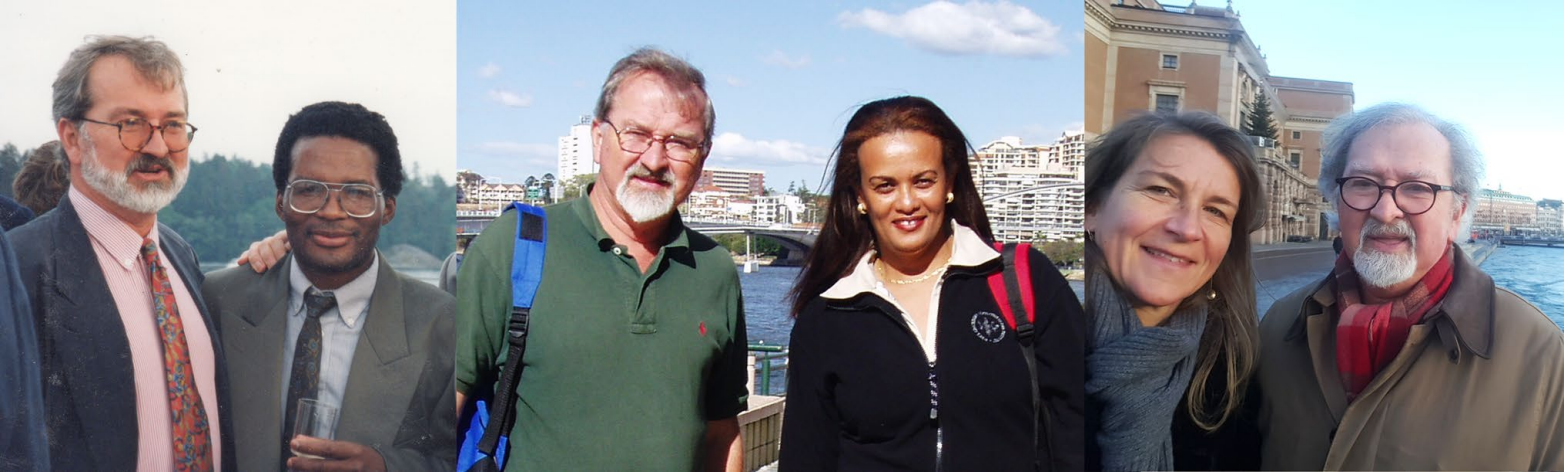


In the wake of January 2024, we mourn the loss of a remarkable individual, Prof. Emeritus Leif Bertilsson. Prof. Bertilsson’s presence was deeply ingrained in the field of pharmacogenetics and personalized medicine. His pioneering research paved the way for transformative advancements, revolutionizing our understanding of how genetic factors influence individual responses to medication. His seminal work not only expanded the frontiers of pharmacogenetic knowledge all over the world but also held the promise of ushering in a new era of tailored therapy, where treatments could be customized to suit the unique pharmacogenetic profile of each patient [1–3]. His departure has cast a shadow over the academic community, leaving behind a profound loss in the field of pharmacogenetics.

This commemorative note collects the personal memory of four of his pupils, who all worked with him, learned from him and committed their work to pharmacogenetics and personalized medicine all over the world.

Julia Stingl wrote: “The first encounter with Leif at an international conference remains vivid. He stood by a poster, discussing the importance of looking at the outliers in analyzing research data—because this was how pharmacogenetic polymorphisms were detected. That moment encapsulated his essence—always eager to share wisdom, always curious, always generous with time. Despite countless achievements and publications, he remained grounded, always willing to engage in meaningful conversations over a beer or a coffee. His emphasis on human diversity was not just academic—it was ingrained in everyday interactions.”

Eleni Aklillu wrote: “I fondly recall Leif’s invaluable influence on many young researchers mentored by him through his pioneering work in the field of pharmacogenetics and clinical pharmacology. His guidance played a pivotal role in shaping our careers. Beyond his scholarly contributions, his warmth and knack for fostering collaborations across continents made him a cherished mentor and friend. Trips taken together, exploring new research avenues while soaking in local culture, remain unforgettable. His laughter and warmth made those experiences truly memorable.”

Collen Masimirembwa wrote: “There are many highlights in my collaborations with Leif over the years, one amazing one is when I invited him to the first pharmacogenetics meeting in Africa conducted in Kenya in 2003. He was so happy to be “home” as he chuckled about Africa being everyone’s home. As usual, everyone loved his jovial nature and humor as he delivered his trademark lecture on the history of pharmacogenetics where he brought all the great names of PGx to life. In our latest collaboration in 2021, in an email, he addressed me as “his academic son,” a huge honor to have such a scientific father. He was a remarkable human being with soul and one of the most accomplished pharmacologists from whom I learnt a lot.”

The Lay Kek wrote: “Prof. Bertilsson’s connection with us extended beyond that initial encounter, as he visited Malaysia on three occasions, gracing us with his wisdom and insights. Each visit was a treasured opportunity for those in the academic community to glean from his vast reservoir of knowledge. His impact on our intellectual landscape was immeasurable, as he shared not only the depth of his expertise but also his passion for fostering a collaborative and inclusive academic environment. As we mourn his loss, let us also celebrate the incredible life he lived and the countless lives he touched along the way.”

An extra and more personal text is added as Digital Supplement.

### Supplementary Information

Below is the link to the electronic supplementary material.

Supplementary file1 (DOCX 138 KB)

## Data Availability

No datasets were generated or analyzed during the current study.
