# Supplementary material for: “Always look at the outliers”: in memoriam Leif Bertilsson
Source: Eur J Clin Pharmacol. 2024 Apr 3;80(7):1111–2. doi: 10.1007/s00228-024-03667-9 (PMC11156748; doi:10.1007/s00228-024-03667-9)
Supplement: Supplementary file 1 — Supplementary file1 (DOCX 138 KB) [file 228_2024_3667_MOESM1_ESM.docx]

**“Always look at the outliers” - in memoriam Leif Bertilsson**

by Julia Stingl^1^(corresponding author), Eleni Aklillu^2^, Collen Masimirembwa^3^, Teh Lay Kek^4^

1. Institute of Clinical Pharmacology, University Hospital RWTH Aachen, Aachen, Germany
2. Global Health Pharmacology and Therapeutics, Department of Global Public Health, Karolinska Institutet, Stockholm, Sweden
3. African Institute of Biomedical Science and Technology, Wilkins Hospital Harare, Harare, Zimbabwe
4. Faculty of Pharmacy, UiTM Puncak Alam Campus, Bandar Puncak Alam, Selangor, Malaysia

It is with profound sadness that we bid farewell to the esteemed Prof Emeritus Leif Bertilsson, a luminary in the academic world, who departed from our midst in January 2024. His passing leaves a void that is felt deeply by all who had the privilege of knowing him, especially those of us fortunate enough to have crossed paths with him in the vibrant tapestry of academia.

This commemorative note is about his achievements in advancing the field of pharmacogenetics and personalized medicine and the way his personality and loveliness left his mark on us, some of his pupils and former PhD students, spreading his legacy all over the world.

**Julia Stingl wrote:**

I met Leif in person at one of my first international conferences, where he was standing in front of a nondescript poster pinned up from several sheets of paper, talking to a very young student from abroad, asking him if he had looked at the distribution of his data and realized that there were several outliers. Looking at these outliers is important, Leif explained and told us, a group of young students standing around this poster, that this is how pharmacogenetics was discovered: There were outliers in drug metabolism who suffered from side effects due to high or extremely high drug exposure or metabolite concentration in the blood [1]{Bertilsson, 1980 #298}{Bertilsson, 1980 #298}. It was later discovered that these outliers belonged to a small subset of carriers of pharmacogenetic variants, and the enzyme exhibiting this variability was termed a polymorphic enzyme [2, 3]. During his five decades of successful research, Leif has elucidated the main genetic background of these pharmacogenetic polymorphisms and described the role of genetic variability in drug metabolism specifically looking at the variability between different populations in the world (some examples from his more than 350 high ranking publications).

Leif dedicated his life to the study of pharmacogenetic variability in patients, with an interest in all kinds of diseases and treatments, but above all - in people. He was interested in the background and life history of every student or researcher who visited him, and he usually kept in touch with his former students for the rest of their lives. He initiated projects to research pharmacogenetics in all parts of the world and loved travelling and chatting over a beer with his research partners, listening carefully to the differences in culture and context of the diseases that made up his particular scientific work. He discovered pharmacogenetic alleles that were only found in certain regions of the world, such as the *17 allele in CYP2D6, which was discovered in African populations and clarified safety issues in drug therapy or complications in the correct dosing of drugs [4-32]. At major international conferences, it was common for a whole group of former students and graduates, who now hold high positions all over the world, to refer to Leif as their old teacher who had taught them how to conduct proper studies and research at a high level to explore the reasons for outliers or deviations from the normal.

He once told me that he wondered what role pharmacogenetics might play in the brain, and that he would expect differences in personality between poor metabolizers and normal metabolizers [33]. He suspected metabolism of endogenous substrates that have neurohumoral activity, such as dopamine or serotonin metabolites, being causal for differences in novelty seeking or risk-taking behavior that is common to the mindset of a scientist [34]. He told me this because he knew that he himself was a poor metabolizer of CYP2D6. In my own research, much later, I discovered differences in psychological phenotypes in the different pharmacogenetic subgroups of CYP2D6 and in the analysis of brain neuroimaging data and behavioral phenotypes, and I saw Leif’s poor metabolizer smiling face in front of me.

I have worked on individualized dosing from a pharmacogenetic point of view, citing and incorporating many studies published by Leif [2, 27, 35-76]. The right dosage - neither too high nor too low - was a central paradigm in his life. Despite his hard work and success in terms of publications and research, he always had time for a chat over a beer, a joke or simply to take the time to study human diversity. I remember once I had to go to the airport and Leif accompanied me - but his flight left many hours later than mine and he could have spent the time in the interesting city instead of at the airport. He told me that he loves working at airports. He then sits at his computer and thinks about science, looking at the people around him - coming from all parts of the world, with all their history and diversity, flying from one part of the world to another. That stress and bustle of people at airports always inspires him to appreciate his own life and the value of his profession as a researcher studying the diversity of people. I don't remember him ever being in a hurry or stressed himself, he enjoyed his life and his profession quietly, and he passed on so much in the process, he was a role model for a generation of researchers from all over the world.

**Eleni Aklillu wrote:**

It is with profound sadness and a heavy heart that I received the news of Leif’s passing. His departure leaves a significant void for those of us who were privileged to be mentored by him and to benefit from his scientific wisdom. The impact he had on the careers of numerous young scientists, myself included, cannot be overstated.

I met Leif for the first time in 1993 at Karolinska Institutet where I did my postgraduate study under the main supervision of Professor Magnus Ingelman-Sundberg and the co-supervision of Leif Bertilsson. Under the mentorship of these two eminent professors, we delved into exploring pharmacogenetic variations in the Sub-Saharan African population. The discovery of CYP2D6 gene duplication in approximately one-third of the Ethiopian population, which we published in 1996, was remarkable [14]. Since then, I have been committed to advancing pharmacogenetic studies in African populations, an area that was largely unexplored at the time. I completed both my master's and PhD training under the co-supervision of Leif and Magnus, and together, we have authored numerous publications [14, 16, 25, 26, 77]. After defending my PhD thesis, I joined Leif’s research group in 2003 and worked with him for over a decade. He introduced me to his extensive international collaborative network spanning Japan, China, Malaysia and in Europe. Together, we conducted several pharmacogenetic and clinical pharmacology research projects and provided joint supervision to several PhD and post-doctoral students from diverse countries [28-32, 78-101].

Leif also introduced me to the field of neuropsychopharmacology and relevance of pharmacogenetics for treatment outcomes. I recall his insight into the potential impact of Khat metabolism by CYP2D6. Khat, a natural amphetamine widely used in East Africa and the Middle East, where CYP2D6 gene duplication occurs at a high frequency. This prompted further research into the importance of genetic variation in Khat-drug interactions[95]. Furthermore, it was Leif's initiative to delve into the hypothesis of CYP1A2 induction by Polycyclic Aromatic Hydrocarbons formed during the roasting of coffee beans. We subsequently confirmed this by the discovery of notable differences in caffeine metabolic ratios between individuals who consume coffee and those who do not [25, 26, 80, 84, 93].

As access to antiretroviral medicines increased in Africa, we promptly initiated a multinational research project aimed at investigating the impact of pharmacogenetic variation in optimizing treatments for infectious diseases such as HIV and tuberculosis. This endeavor was particularly important given the unique genetic and environmental diversity among black Africans compared to other populations. Our journey to Ethiopia and Tanzania during the Pharmagene TB-HIV project was truly memorable, blending scientific discovery with rich social experiences. The cherished memories of our safari in Ngorongoro National Park and the enchanting exploration of Zanzibar remain vivid. Leif's “White African” humor added an extra layer of warmth and camaraderie, making the experience truly special.

Leif was not only a brilliant scientist but also a generous mentor and friend. Our journeys to various conferences provided unforgettable experiences as we met with friends and fellow scientists. I cherish fond memories of our travels to Australia, Japan, Tanzania, and Ethiopia, where we had the opportunity to connect with our students and collaborators. His guidance and support were instrumental in shaping my scientific path, and I am eternally grateful for his mentorship. His wisdom has left an enduring mark on the scientific growth of many young researchers, including myself. Leif, my friend, may your soul find eternal peace. Your impact will be everlasting.

**Collen Masimirembwa wrote:**

I met Leif in 1993 where he was a member of my PhD supervisory group that included Prof. Magnus Ingelman-Sundberg and Prof. Folke Sjöqvist. From the publication of our first paper in 1996 when we discovered the African specific variant, CYP2D6*17 [15], to our last paper in 2021 where we evaluated the possible use of 1-hydroxylation of deoxycholic acid as a non-invasive urinary biomarker of CYP3A activity {Li, 2021 #2}, the evolution from a supervisor, to a mentor to a friend was fluid and gratifying. His best quality was to see the best in everyone, he could talk as if from a reference manual about the historical development of PGx over lunch, of the various studies he and his colleagues had conducted that had led us to the current findings in the field, always being generous when it came to giving credit to other scientists. Always jovial of spirit and full of new ideas of what studies could be done to address new challenges in the field, ‘ Collen, I think we are yet to find amazing things in the populations of Africa’ he would say with excitement as we analyzed data from the many studies we did together . His emphasis on the importance of the phenotype always balancing others’ love for genetic variation, a collaboration of giants in science that resulted in outstanding pharmacogenetic research at the Karolinska institute.

There are many highlights in our collaborations over the years, one such amazing experience was when I invited him to the first PGx meeting in Africa conducted in Kenya in 2003. He was so happy to be ‘home’ as he chuckled about Africa being everyone’s home. As usual, everyone loved his jovial nature and humor as he delivered his trademark lecture on the history of pharmacogenetics where he brought all the great names of PGx to life.

On one hand we are sad at the loss of a legend, on the other we also celebrate the life of a man who exuded a ‘Buddha-like’ serenity. May his soul rest in peace in the knowledge of the great wealth of knowledge he has passed on to the next generation of pharmacogenetics researchers. In our latest collaboration in 2021, in an email he addressed me as ‘his academic son,’ a huge honor to have such a scientific father. He was a remarkable human being with soul and one of the most accomplished pharmacologists from whom I learnt a lot.

**Teh Lay Kek wrote:**

I had the distinct honor of encountering Prof Bertilsson approximately two decades ago during a seminar held in Nigeria. Even then, his intellectual prowess and unwavering commitment to the pursuit of knowledge were evident. What set him apart was not only his exceptional scholarly contributions but also the genuine warmth with which he engaged and inspired everyone around him.

Prof Bertilsson's connection with us extended beyond that initial encounter, as he visited Malaysia on three occasions, gracing us with his wisdom and insights. Each visit was a treasured opportunity for those in the academic community to glean from his vast reservoir of knowledge. His impact on our intellectual landscape was immeasurable, as he shared not only the depth of his expertise but also his passion for fostering a collaborative and inclusive academic environment.

The memories of those interactions in Malaysia remain etched in our minds, serving as a testament to the transcendent nature of Prof Bertilsson's influence. The exchange of ideas during those visits enriched our understanding and fueled our collective pursuit of excellence.

As we reflect on Prof Bertilsson's contributions, let us not only celebrate the scholarly legacy he leaves behind but also the personal connections he forged. His warmth, humility, and genuine interest in the growth of others endeared him to many. The impact of his teachings, both inside and outside the lecture hall, resonates in the careers and lives of those who were fortunate enough to learn from him.

In honoring Prof Emeritus Leif Bertilsson, let us carry forward the torch of knowledge that he so passionately ignited during his visits to Malaysia and beyond. May his legacy inspire future generations to approach academia with the same fervor, generosity, and collaborative spirit that defined his illustrious career.

Though he may no longer be physically present, Prof Bertilsson's legacy lives on in the collective memory of the academic community he touched. In this time of mourning, let us extend our deepest condolences to his family, friends, and colleagues. Rest in peace, Prof Emeritus Leif Bertilsson, knowing that your brilliance and benevolence will forever resonate in the corridors of learning and in the hearts of those privileged to have known you.

**References**

1 Bertilsson L, Dengler HJ, Eichelbaum M, Schulz HU (1980) Pharmacogenetic covariation of defective N-oxidation of sparteine and 4-hydroxylation of debrisoquine. Eur J Clin Pharmacol 17 (2): 153-155 DOI 10.1007/BF00562624

2 Sjoqvist F, Bertilsson L (1986) Slow hydroxylation of tricyclic antidepressants--relationship to polymorphic drug oxidation. Prog Clin Biol Res 214: 169-188

3 Brosen K, Gram LF, Haghfelt T, Bertilsson L (1987) Extensive metabolizers of debrisoquine become poor metabolizers during quinidine treatment. Pharmacol Toxicol 60 (4): 312-314 DOI 10.1111/j.1600-0773.1987.tb01758.x

4 Steiner E, Bertilsson L, Sawe J, Bertling I, Sjoqvist F (1988) Polymorphic debrisoquin hydroxylation in 757 Swedish subjects. Clin Pharmacol Ther 44 (4): 431-435 DOI 10.1038/clpt.1988.176

5 Andersson T, Regardh CG, Dahl-Puustinen ML, Bertilsson L (1990) Slow omeprazole metabolizers are also poor S-mephenytoin hydroxylators. Ther Drug Monit 12 (4): 415-416 DOI 10.1097/00007691-199007000-00020

6 Zhang YA, Reviriego J, Lou YQ, Sjoqvist F, Bertilsson L (1990) Diazepam metabolism in native Chinese poor and extensive hydroxylators of S-mephenytoin: interethnic differences in comparison with white subjects. Clin Pharmacol Ther 48 (5): 496-502 DOI 10.1038/clpt.1990.185

7 Johansson I, Yue QY, Dahl ML, Heim M, Sawe J, Bertilsson L, Meyer UA, Sjoqvist F, Ingelman-Sundberg M (1991) Genetic analysis of the interethnic difference between Chinese and Caucasians in the polymorphic metabolism of debrisoquine and codeine. Eur J Clin Pharmacol 40 (6): 553-556 DOI 10.1007/BF00279968

8 Dahl ML, Johansson I, Yue QY, Sawe J, Bertilsson L, Ingelman-Sundberg M, Sjoqvist F (1992) Interethnic differences in drug oxidation. Implications for the utilization of antidepressants and neuroleptics. Clin Neuropharmacol 15 Suppl 1 Pt A: 88A-89A DOI 10.1097/00002826-199201001-00047

9 Ingelman-Sundberg M, Johansson I, Persson I, Yue QY, Dahl ML, Bertilsson L, Sjoqvist F (1992) Genetic polymorphism of cytochromes P450: interethnic differences and relationship to incidence of lung cancer. Pharmacogenetics 2 (6): 264-271 DOI 10.1097/00008571-199212000-00004

10 Reviriego J, Bertilsson L, Carrillo JA, Llerena A, Valdivielso MJ, Benitez J (1993) Frequency of S-mephenytoin hydroxylation deficiency in 373 Spanish subjects compared to other Caucasian populations. Eur J Clin Pharmacol 44 (6): 593-595 DOI 10.1007/BF02440867

11 Bertilsson L (1995) Geographical/interracial differences in polymorphic drug oxidation. Current state of knowledge of cytochromes P450 (CYP) 2D6 and 2C19. Clin Pharmacokinet 29 (3): 192-209 DOI 10.2165/00003088-199529030-00005

12 Dahl ML, Yue QY, Roh HK, Johansson I, Sawe J, Sjoqvist F, Bertilsson L (1995) Genetic analysis of the CYP2D locus in relation to debrisoquine hydroxylation capacity in Korean, Japanese and Chinese subjects. Pharmacogenetics 5 (3): 159-164 DOI 10.1097/00008571-199506000-00004

13 Masimirembwa C, Bertilsson L, Johansson I, Hasler JA, Ingelman-Sundberg M (1995) Phenotyping and genotyping of S-mephenytoin hydroxylase (cytochrome P450 2C19) in a Shona population of Zimbabwe. Clin Pharmacol Ther 57 (6): 656-661 DOI 10.1016/0009-9236(95)90228-7

14 Aklillu E, Persson I, Bertilsson L, Johansson I, Rodrigues F, Ingelman-Sundberg M (1996) Frequent distribution of ultrarapid metabolizers of debrisoquine in an ethiopian population carrying duplicated and multiduplicated functional CYP2D6 alleles. J Pharmacol Exp Ther 278 (1): 441-446

15 Masimirembwa C, Persson I, Bertilsson L, Hasler J, Ingelman-Sundberg M (1996) A novel mutant variant of the CYP2D6 gene (CYP2D6*17) common in a black African population: association with diminished debrisoquine hydroxylase activity. Br J Clin Pharmacol 42 (6): 713-719 DOI 10.1046/j.1365-2125.1996.00489.x

16 Persson I, Aklillu E, Rodrigues F, Bertilsson L, Ingelman-Sundberg M (1996) S-mephenytoin hydroxylation phenotype and CYP2C19 genotype among Ethiopians. Pharmacogenetics 6 (6): 521-526 DOI 10.1097/00008571-199612000-00005

17 Roh HK, Dahl ML, Johansson I, Ingelman-Sundberg M, Cha YN, Bertilsson L (1996) Debrisoquine and S-mephenytoin hydroxylation phenotypes and genotypes in a Korean population. Pharmacogenetics 6 (5): 441-447 DOI 10.1097/00008571-199610000-00008

18 Roh HK, Dahl ML, Tybring G, Yamada H, Cha YN, Bertilsson L (1996) CYP2C19 genotype and phenotype determined by omeprazole in a Korean population. Pharmacogenetics 6 (6): 547-551 DOI 10.1097/00008571-199612000-00008

19 Herrlin K, Massele AY, Jande M, Alm C, Tybring G, Abdi YA, Wennerholm A, Johansson I, Dahl ML, Bertilsson L, Gustafsson LL (1998) Bantu Tanzanians have a decreased capacity to metabolize omeprazole and mephenytoin in relation to their CYP2C19 genotype. Clin Pharmacol Ther 64 (4): 391-401 DOI 10.1016/S0009-9236(98)90070-4

20 Kaneko A, Lum JK, Yaviong L, Takahashi N, Ishizaki T, Bertilsson L, Kobayakawa T, Bjorkman A (1999) High and variable frequencies of CYP2C19 mutations: medical consequences of poor drug metabolism in Vanuatu and other Pacific islands. Pharmacogenetics 9 (5): 581-590

21 Shimoda K, Jerling M, Bottiger Y, Yasuda S, Morita S, Bertilsson L (1999) Pronounced differences in the dispositon of clomipramine between Japanese and Swedish patients. J Clin Psychopharmacol 19 (5): 393-400 DOI 10.1097/00004714-199910000-00002

22 Wennerholm A, Johansson I, Massele AY, Lande M, Alm C, Aden-Abdi Y, Dahl ML, Ingelman-Sundberg M, Bertilsson L, Gustafsson LL (1999) Decreased capacity for debrisoquine metabolism among black Tanzanians: analyses of the CYP2D6 genotype and phenotype. Pharmacogenetics 9 (6): 707-714

23 Roh HK, Kim CE, Chung WG, Park CS, Svensson JO, Bertilsson L (2001) Risperidone metabolism in relation to CYP2D6*10 allele in Korean schizophrenic patients. Eur J Clin Pharmacol 57 (9): 671-675 DOI 10.1007/s002280100372

24 Wennerholm A, Johansson I, Hidestrand M, Bertilsson L, Gustafsson LL, Ingelman-Sundberg M (2001) Characterization of the CYP2D6*29 allele commonly present in a black Tanzanian population causing reduced catalytic activity. Pharmacogenetics 11 (5): 417-427 DOI 10.1097/00008571-200107000-00005

25 Aklillu E, Carrillo JA, Makonnen E, Bertilsson L, Ingelman-Sundberg M (2003) Xanthine oxidase activity is influenced by environmental factors in Ethiopians. Eur J Clin Pharmacol 59 (7): 533-536 DOI 10.1007/s00228-003-0653-8

26 Aklillu E, Carrillo JA, Makonnen E, Hellman K, Pitarque M, Bertilsson L, Ingelman-Sundberg M (2003) Genetic polymorphism of CYP1A2 in Ethiopians affecting induction and expression: characterization of novel haplotypes with single-nucleotide polymorphisms in intron 1. Mol Pharmacol 64 (3): 659-669 DOI 10.1124/mol.64.3.659

27 Kirchheiner J, Bertilsson L, Bruus H, Wolff A, Roots I, Bauer M (2003) Individualized medicine - implementation of pharmacogenetic diagnostics in antidepressant drug treatment of major depressive disorders. Pharmacopsychiatry 36 Suppl 3: S235-243 DOI 10.1055/s-2003-45136

28 Mirghani RA, Sayi J, Aklillu E, Allqvist A, Jande M, Wennerholm A, Eriksen J, Herben VM, Jones BC, Gustafsson LL, Bertilsson L (2006) CYP3A5 genotype has significant effect on quinine 3-hydroxylation in Tanzanians, who have lower total CYP3A activity than a Swedish population. Pharmacogenet Genomics 16 (9): 637-645 DOI 10.1097/01.fpc.0000230411.89973.1b

29 Djordjevic N, Carrillo JA, van den Broek MP, Kishikawa J, Roh HK, Bertilsson L, Aklillu E (2013) Comparisons of CYP2A6 genotype and enzyme activity between Swedes and Koreans. Drug Metab Pharmacokinet 28 (2): 93-97 DOI 10.2133/dmpk.dmpk-12-rg-029

30 Ngaimisi E, Habtewold A, Minzi O, Makonnen E, Mugusi S, Amogne W, Yimer G, Riedel KD, Janabi M, Aderaye G, Mugusi F, Bertilsson L, Aklillu E, Burhenne J (2013) Importance of ethnicity, CYP2B6 and ABCB1 genotype for efavirenz pharmacokinetics and treatment outcomes: a parallel-group prospective cohort study in two sub-Saharan Africa populations. PLoS One 8 (7): e67946 DOI 10.1371/journal.pone.0067946

31 Hatta FH, Lundblad M, Ramsjo M, Kang JH, Roh HK, Bertilsson L, Eliasson E, Aklillu E (2015) Differences in CYP2C9 Genotype and Enzyme Activity Between Swedes and Koreans of Relevance for Personalized Medicine: Role of Ethnicity, Genotype, Smoking, Age, and Sex. OMICS 19 (6): 346-353 DOI 10.1089/omi.2015.0022

32 Aklillu E, Carrillo JA, Makonnen E, Bertilsson L, Djordjevic N (2018) N-Acetyltransferase-2 (NAT2) phenotype is influenced by genotype-environment interaction in Ethiopians. Eur J Clin Pharmacol 74 (7): 903-911 DOI 10.1007/s00228-018-2448-y

33 Bertilsson L, Alm C, De Las Carreras C, Widen J, Edman G, Schalling D (1989) Debrisoquine hydroxylation polymorphism and personality. Lancet 1 (8637): 555 DOI 10.1016/s0140-6736(89)90094-9

34 Llerena A, Edman G, Cobaleda J, Benitez J, Schalling D, Bertilsson L (1993) Relationship between personality and debrisoquine hydroxylation capacity. Suggestion of an endogenous neuroactive substrate or product of the cytochrome P4502D6. Acta Psychiatr Scand 87 (1): 23-28 DOI 10.1111/j.1600-0447.1993.tb03325.x

35 Alexanderson B, Bertilsson L, Borga O, Sjoqvist F (1971) Studies on the metabolism and pharamcokinetics of nortriptyline and desmethylimipramine in man. Chem Biol Interact 3 (4): 235-236 DOI 10.1016/0009-2797(71)90039-1

36 Sjoqvist F, Alexanderson B, Asberg M, Bertilsson L, Borga O, Hamberger B, Tuck D (1971) Pharmacokinetics and biological effects of nortriptyline in man. Acta Pharmacol Toxicol (Copenh) 29 Suppl 3: 255-280 DOI 10.1111/j.1600-0773.1971.tb03306.x

37 Asberg M, Thoren P, Traskman L, Bertilsson L, Ringberger V (1976) "Serotonin depression"--a biochemical subgroup within the affective disorders? Science 191 (4226): 478-480 DOI 10.1126/science.1246632

38 Mellstrom B, Bertilsson L, Traskman L, Rollins D, Asberg M, Sjoqvist F (1979) Intraindividual similarity in the metabolism of amitriptyline and chlorimipramine in depressed patients. Pharmacology 19 (5): 282-287 DOI 10.1159/000137324

39 Traskman L, Asberg M, Bertilsson L, Cronholm B, Mellstrom B, Neckers LM, Sjoqvist F, Thoren P, Tybring G (1979) Plasma levels of chlorimipramine and its demethyl metabolite during treatment of depression. Clin Pharmacol Ther 26 (5): 600-610 DOI 10.1002/cpt1979265600

40 Bertilsson L, Eichelbaum M, Mellstrom B, Sawe J, Schulz HU, Sjoqvist F (1980) Nortriptyline and antipyrine clearance in relation to debrisoquine hydroxylation in man. Life Sci 27 (18): 1673-1677 DOI 10.1016/0024-3205(80)90642-6

41 Rollins DE, Alvan G, Bertilsson L, Gillette JR, Mellstrom B, Sjoqvist F, Traskman L (1980) Interindividual differences in amitriptyline demethylation. Clin Pharmacol Ther 28 (1): 121-129 DOI 10.1038/clpt.1980.140

42 Sjoqvist F, Bertilsson L, Asberg M (1980) Monitoring tricyclic antidepressants. Ther Drug Monit 2 (1): 85-93 DOI 10.1097/00007691-198001000-00010

43 Bertilsson L, Mellstrom B, Sjokvist F, Martenson B, Asberg M (1981) Slow hydroxylation of nortriptyline and concomitant poor debrisoquine hydroxylation: clinical implications. Lancet 1 (8219): 560-561 DOI 10.1016/s0140-6736(81)92894-4

44 Mellstrom B, Bertilsson L, Sawe J, Schulz HU, Sjoqvist F (1981) E- and Z-10-hydroxylation of nortriptyline: relationship to polymorphic debrisoquine hydroxylation. Clin Pharmacol Ther 30 (2): 189-193 DOI 10.1038/clpt.1981.147

45 Traskman-Bendz L, Asberg M, Bertilsson L (1981) Serotonin and noradrenaline uptake inhibitors in the treatment of depression--relationship to 5-HIAA in spinal fluid. Acta Psychiatr Scand Suppl 290: 209-218 DOI 10.1111/j.1600-0447.1981.tb00722.x

46 Mellstrom B, Alvan G, Bertilsson L, Potter WZ, Sawe J, Sjoqvist F (1982) Nortriptyline formation after single oral and intramuscular doses of amitriptyline. Clin Pharmacol Ther 32 (5): 664-667 DOI 10.1038/clpt.1982.219

47 Mellstrom B, Bertilsson L, Birgersson C, Goransson M, von Bahr C (1983) E- and Z-10-hydroxylation of nortriptyline by human liver microsomes--methods and characterization. Drug Metab Dispos 11 (2): 115-119

48 Mellstrom B, Bertilsson L, Lou YC, Sawe J, Sjoqvist F (1983) Amitriptyline metabolism: relationship to polymorphic debrisoquine hydroxylation. Clin Pharmacol Ther 34 (4): 516-520 DOI 10.1038/clpt.1983.207

49 Sjoqvist F, Bertilsson L (1984) Clinical pharmacology of antidepressant drugs: pharmacogenetics. Adv Biochem Psychopharmacol 39: 359-372

50 Nordin C, Siwers B, Benitez J, Bertilsson L (1985) Plasma concentrations of nortriptyline and its 10-hydroxy metabolite in depressed patients--relationship to the debrisoquine hydroxylation metabolic ratio. Br J Clin Pharmacol 19 (6): 832-835 DOI 10.1111/j.1365-2125.1985.tb02723.x

51 Bertilsson L, Nordin C, Otani K, Resul B, Scheinin M, Siwers B, Sjoqvist F (1986) Disposition of single oral doses of E-10-hydroxynortriptyline in healthy subjects, with some observations on pharmacodynamic effects. Clin Pharmacol Ther 40 (3): 261-267 DOI 10.1038/clpt.1986.173

52 Brosen K, Klysner R, Gram LF, Otton SV, Bech P, Bertilsson L (1986) Steady-state concentrations of imipramine and its metabolites in relation to the sparteine/debrisoquine polymorphism. Eur J Clin Pharmacol 30 (6): 679-684 DOI 10.1007/BF00608215

53 Mellstrom B, Sawe J, Bertilsson L, Sjoqvist F (1986) Amitriptyline metabolism: association with debrisoquin hydroxylation in nonsmokers. Clin Pharmacol Ther 39 (4): 369-371 DOI 10.1038/clpt.1986.56

54 Malmgren R, Aberg-Wistedt A, Bertilsson L (1987) Serotonin uptake inhibition during treatment of depression with nortriptyline caused by parent drug and not by 10-hydroxymetabolites. Psychopharmacology (Berl) 92 (2): 169-172 DOI 10.1007/BF00177910

55 Nordin C, Bertilsson L, Otani K, Widmark A (1987) Little anticholinergic effect of E-10-hydroxynortriptyline compared with nortriptyline in healthy subjects. Clin Pharmacol Ther 41 (1): 97-102 DOI 10.1038/clpt.1987.16

56 Nordin C, Bertilsson L, Siwers B (1987) Clinical and biochemical effects during treatment of depression with nortriptyline: the role of 10-hydroxynortriptyline. Clin Pharmacol Ther 42 (1): 10-19 DOI 10.1038/clpt.1987.101

57 Wagner A, Aberg-Wistedt A, Asberg M, Bertilsson L, Martensson B, Montero D (1987) Effects of antidepressant treatments on platelet tritiated imipramine binding in major depressive disorder. Arch Gen Psychiatry 44 (10): 870-877 DOI 10.1001/archpsyc.1987.01800220032006

58 Bertilsson L, Dahl-Puustinen ML, Nordin C (1989) E-10-hydroxynortriptyline: effects and disposition of a potential novel antidepressant. Psychopharmacol Ser 7: 52-59 DOI 10.1007/978-3-642-74430-3_6

59 Martensson B, Nyberg S, Toresson G, Brodin E, Bertilsson L (1989) Fluoxetine treatment of depression. Clinical effects, drug concentrations and monoamine metabolites and N-terminally extended substance P in cerebrospinal fluid. Acta Psychiatr Scand 79 (6): 586-596 DOI 10.1111/j.1600-0447.1989.tb10307.x

60 Nordin C, Bertilsson L, Dahl ML, Resul B, Toresson G, Sjoqvist F (1991) Treatment of depression with E-10-hydroxynortriptyline--a pilot study on biochemical effects and pharmacokinetics. Psychopharmacology (Berl) 103 (3): 287-290 DOI 10.1007/BF02244280

61 Dahl ML, Tybring G, Elwin CE, Alm C, Andreasson K, Gyllenpalm M, Bertilsson L (1994) Stereoselective disposition of mianserin is related to debrisoquin hydroxylation polymorphism. Clin Pharmacol Ther 56 (2): 176-183 DOI 10.1038/clpt.1994.121

62 Tybring G, Otani K, Kaneko S, Mihara K, Fukushima Y, Bertilsson L (1995) Enantioselective determination of mianserin and its desmethyl metabolite in plasma during treatment of depressed Japanese patients. Ther Drug Monit 17 (5): 516-521 DOI 10.1097/00007691-199510000-00013

63 Carrillo JA, Dahl ML, Svensson JO, Alm C, Rodriguez I, Bertilsson L (1996) Disposition of fluvoxamine in humans is determined by the polymorphic CYP2D6 and also by the CYP1A2 activity. Clin Pharmacol Ther 60 (2): 183-190 DOI 10.1016/S0009-9236(96)90134-4

64 Meyer UA, Amrein R, Balant LP, Bertilsson L, Eichelbaum M, Guentert TW, Henauer S, Jackson P, Laux G, Mikkelsen H, Peck C, Pollock BG, Priest R, Sjoqvist F, Delini-Stula A (1996) Antidepressants and drug-metabolizing enzymes--expert group report. Acta Psychiatr Scand 93 (2): 71-79 DOI 10.1111/j.1600-0447.1996.tb09805.x

65 Bertilsson L, Dahl ML, Tybring G (1997) Pharmacogenetics of antidepressants: clinical aspects. Acta Psychiatr Scand Suppl 391: 14-21 DOI 10.1111/j.1600-0447.1997.tb05954.x

66 Mihara K, Otani K, Tybring G, Dahl ML, Bertilsson L, Kaneko S (1997) The CYP2D6 genotype and plasma concentrations of mianserin enantiomers in relation to therapeutic response to mianserin in depressed Japanese patients. J Clin Psychopharmacol 17 (6): 467-471 DOI 10.1097/00004714-199712000-00005

67 Shimoda K, Yasuda S, Morita S, Shibasaki M, Someya T, Bertilsson L, Takahashi S (1997) Significance of monitoring plasma levels of amitriptyline, and its hydroxylated and desmethylated metabolites in prediction of the clinical outcome of depressive state. Psychiatry Clin Neurosci 51 (1): 35-41 DOI 10.1111/j.1440-1819.1997.tb02364.x

68 Lidberg L, Belfrage H, Bertilsson L, Evenden MM, Asberg M (2000) Suicide attempts and impulse control disorder are related to low cerebrospinal fluid 5-HIAA in mentally disordered violent offenders. Acta Psychiatr Scand 101 (5): 395-402 DOI 10.1034/j.1600-0447.2000.101005395.x

69 von Bahr C, Ursing C, Yasui N, Tybring G, Bertilsson L, Rojdmark S (2000) Fluvoxamine but not citalopram increases serum melatonin in healthy subjects-- an indication that cytochrome P450 CYP1A2 and CYP2C19 hydroxylate melatonin. Eur J Clin Pharmacol 56 (2): 123-127 DOI 10.1007/s002280050729

70 Laine K, Tybring G, Hartter S, Andersson K, Svensson JO, Widen J, Bertilsson L (2001) Inhibition of cytochrome P4502D6 activity with paroxetine normalizes the ultrarapid metabolizer phenotype as measured by nortriptyline pharmacokinetics and the debrisoquin test. Clin Pharmacol Ther 70 (4): 327-335

71 Bertilsson L, Dahl ML, Dalen P, Al-Shurbaji A (2002) Molecular genetics of CYP2D6: clinical relevance with focus on psychotropic drugs. Br J Clin Pharmacol 53 (2): 111-122 DOI 10.1046/j.0306-5251.2001.01548.x

72 Herrlin K, Yasui-Furukori N, Tybring G, Widen J, Gustafsson LL, Bertilsson L (2003) Metabolism of citalopram enantiomers in CYP2C19/CYP2D6 phenotyped panels of healthy Swedes. Br J Clin Pharmacol 56 (4): 415-421 DOI 10.1046/j.1365-2125.2003.01874.x

73 Kawanishi C, Lundgren S, Agren H, Bertilsson L (2004) Increased incidence of CYP2D6 gene duplication in patients with persistent mood disorders: ultrarapid metabolism of antidepressants as a cause of nonresponse. A pilot study. Eur J Clin Pharmacol 59 (11): 803-807 DOI 10.1007/s00228-003-0701-4

74 Laine K, Kytola J, Bertilsson L (2004) Severe adverse effects in a newborn with two defective CYP2D6 alleles after exposure to paroxetine during late pregnancy. Ther Drug Monit 26 (6): 685-687 DOI 10.1097/00007691-200412000-00016

75 Bertilsson L (2007) Metabolism of antidepressant and neuroleptic drugs by cytochrome p450s: clinical and interethnic aspects. Clin Pharmacol Ther 82 (5): 606-609 DOI 10.1038/sj.clpt.6100358

76 Ohlsson Rosenborg S, Mwinyi J, Andersson M, Baldwin RM, Pedersen RS, Sim SC, Bertilsson L, Ingelman-Sundberg M, Eliasson E (2008) Kinetics of omeprazole and escitalopram in relation to the CYP2C19*17 allele in healthy subjects. Eur J Clin Pharmacol 64 (12): 1175-1179 DOI 10.1007/s00228-008-0529-z

77 Aklillu E, Herrlin K, Gustafsson LL, Bertilsson L, Ingelman-Sundberg M (2002) Evidence for environmental influence on CYP2D6-catalysed debrisoquine hydroxylation as demonstrated by phenotyping and genotyping of Ethiopians living in Ethiopia or in Sweden. Pharmacogenetics 12 (5): 375-383 DOI 10.1097/00008571-200207000-00005

78 Ozdemir V, Aklillu E, Mee S, Bertilsson L, Albers LJ, Graham JE, Caligiuri M, Lohr JB, Reist C (2006) Pharmacogenetics for off-patent antipsychotics: reframing the risk for tardive dyskinesia and access to essential medicines. Expert Opin Pharmacother 7 (2): 119-133 DOI 10.1517/14656566.7.2.119

79 Sim SC, Risinger C, Dahl ML, Aklillu E, Christensen M, Bertilsson L, Ingelman-Sundberg M (2006) A common novel CYP2C19 gene variant causes ultrarapid drug metabolism relevant for the drug response to proton pump inhibitors and antidepressants. Clin Pharmacol Ther 79 (1): 103-113 DOI 10.1016/j.clpt.2005.10.002

80 Ghotbi R, Christensen M, Roh HK, Ingelman-Sundberg M, Aklillu E, Bertilsson L (2007) Comparisons of CYP1A2 genetic polymorphisms, enzyme activity and the genotype-phenotype relationship in Swedes and Koreans. Eur J Clin Pharmacol 63 (6): 537-546 DOI 10.1007/s00228-007-0288-2

81 Josephson F, Allqvist A, Janabi M, Sayi J, Aklillu E, Jande M, Mahindi M, Burhenne J, Bottiger Y, Gustafsson LL, Haefeli WE, Bertilsson L (2007) CYP3A5 genotype has an impact on the metabolism of the HIV protease inhibitor saquinavir. Clin Pharmacol Ther 81 (5): 708-712 DOI 10.1038/sj.clpt.6100117

82 Kishida I, Aklillu E, Kawanishi C, Bertilsson L, Agren H (2007) Monoamine metabolites level in CSF is related to the 5-HTT gene polymorphism in treatment-resistant depression. Neuropsychopharmacology 32 (10): 2143-2151 DOI 10.1038/sj.npp.1301336

83 Diczfalusy U, Miura J, Roh HK, Mirghani RA, Sayi J, Larsson H, Bodin KG, Allqvist A, Jande M, Kim JW, Aklillu E, Gustafsson LL, Bertilsson L (2008) 4Beta-hydroxycholesterol is a new endogenous CYP3A marker: relationship to CYP3A5 genotype, quinine 3-hydroxylation and sex in Koreans, Swedes and Tanzanians. Pharmacogenet Genomics 18 (3): 201-208 DOI 10.1097/FPC.0b013e3282f50ee9

84 Djordjevic N, Ghotbi R, Bertilsson L, Jankovic S, Aklillu E (2008) Induction of CYP1A2 by heavy coffee consumption in Serbs and Swedes. Eur J Clin Pharmacol 64 (4): 381-385 DOI 10.1007/s00228-007-0438-6

85 Ghotbi R, Gomez A, Milani L, Tybring G, Syvanen AC, Bertilsson L, Ingelman-Sundberg M, Aklillu E (2009) Allele-specific expression and gene methylation in the control of CYP1A2 mRNA level in human livers. Pharmacogenomics J 9 (3): 208-217 DOI 10.1038/tpj.2009.4

86 Hilli J, Heikkinen T, Rontu R, Lehtimaki T, Kishida I, Aklillu E, Bertilsson L, Vahlberg T, Laine K (2009) MAO-A and COMT genotypes as possible regulators of perinatal serotonergic symptoms after in utero exposure to SSRIs. Eur Neuropsychopharmacol 19 (5): 363-370 DOI 10.1016/j.euroneuro.2009.01.006

87 Ghotbi R, Mannheimer B, Aklillu E, Suda A, Bertilsson L, Eliasson E, Osby U (2010) Carriers of the UGT1A4 142T>G gene variant are predisposed to reduced olanzapine exposure--an impact similar to male gender or smoking in schizophrenic patients. Eur J Clin Pharmacol 66 (5): 465-474 DOI 10.1007/s00228-009-0783-8

88 Ramsjo M, Aklillu E, Bohman L, Ingelman-Sundberg M, Roh HK, Bertilsson L (2010) CYP2C19 activity comparison between Swedes and Koreans: effect of genotype, sex, oral contraceptive use, and smoking. Eur J Clin Pharmacol 66 (9): 871-877 DOI 10.1007/s00228-010-0835-0

89 Ngaimisi E, Mugusi S, Minzi O, Sasi P, Riedel KD, Suda A, Ueda N, Janabi M, Mugusi F, Haefeli WE, Bertilsson L, Burhenne J, Aklillu E (2011) Effect of rifampicin and CYP2B6 genotype on long-term efavirenz autoinduction and plasma exposure in HIV patients with or without tuberculosis. Clin Pharmacol Ther 90 (3): 406-413 DOI 10.1038/clpt.2011.129

90 Djordjevic N, Carrillo JA, Roh HK, Karlsson S, Ueda N, Bertilsson L, Aklillu E (2012) Comparison of N-acetyltransferase-2 enzyme genotype-phenotype and xanthine oxidase enzyme activity between Swedes and Koreans. J Clin Pharmacol 52 (10): 1527-1534 DOI 10.1177/0091270011420261

91 Hatta FH, Teh LK, Hellden A, Hellgren KE, Roh HK, Salleh MZ, Aklillu E, Bertilsson L (2012) Search for the molecular basis of ultra-rapid CYP2C9-catalysed metabolism: relationship between SNP IVS8-109A>T and the losartan metabolism phenotype in Swedes. Eur J Clin Pharmacol 68 (7): 1033-1042 DOI 10.1007/s00228-012-1210-0

92 Habtewold A, Amogne W, Makonnen E, Yimer G, Nylen H, Riedel KD, Aderaye G, Bertilsson L, Burhenne J, Diczfalusy U, Aklillu E (2013) Pharmacogenetic and pharmacokinetic aspects of CYP3A induction by efavirenz in HIV patients. Pharmacogenomics J 13 (6): 484-489 DOI 10.1038/tpj.2012.46

93 Aklillu E, Djordjevic N, Carrillo JA, Makonnen E, Bertilsson L, Ingelman-Sundberg M (2014) High CYP2A6 enzyme activity as measured by a caffeine test and unique distribution of CYP2A6 variant alleles in Ethiopian population. OMICS 18 (7): 446-453 DOI 10.1089/omi.2013.0140

94 Ngaimisi E, Minzi O, Mugusi S, Sasi P, Riedel KD, Suda A, Ueda N, Bakari M, Janabi M, Mugusi F, Bertilsson L, Burhenne J, Aklillu E, Diczfalusy U (2014) Pharmacokinetic and pharmacogenomic modelling of the CYP3A activity marker 4beta-hydroxycholesterol during efavirenz treatment and efavirenz/rifampicin co-treatment. J Antimicrob Chemother 69 (12): 3311-3319 DOI 10.1093/jac/dku286

95 Bedada W, de Andres F, Engidawork E, Pohanka A, Beck O, Bertilsson L, Llerena A, Aklillu E (2015) The Psychostimulant Khat (Catha edulis) Inhibits CYP2D6 Enzyme Activity in Humans. J Clin Psychopharmacol 35 (6): 694-699 DOI 10.1097/JCP.0000000000000413

96 Habtewold A, Makonnen E, Amogne W, Yimer G, Aderaye G, Bertilsson L, Burhenne J, Aklillu E (2015) Is there a need to increase the dose of efavirenz during concomitant rifampicin-based antituberculosis therapy in sub-Saharan Africa? The HIV-TB pharmagene study. Pharmacogenomics 16 (10): 1047-1064 DOI 10.2217/pgs.15.35

97 Habtewold A, Aklillu E, Makonnen E, Amogne W, Yimer G, Aderaye G, Bertilsson L, Owen JS, Burhenne J (2016) Long-Term Effect of Rifampicin-Based Anti-TB Regimen Coadministration on the Pharmacokinetic Parameters of Efavirenz and 8-Hydroxy-Efavirenz in Ethiopian Patients. J Clin Pharmacol 56 (12): 1538-1549 DOI 10.1002/jcph.756

98 Nylen H, Habtewold A, Makonnen E, Yimer G, Bertilsson L, Burhenne J, Diczfalusy U, Aklillu E (2016) Prevalence and risk factors for efavirenz-based antiretroviral treatment-associated severe vitamin D deficiency: A prospective cohort study. Medicine (Baltimore) 95 (34): e4631 DOI 10.1097/MD.0000000000004631

99 Habtewold A, Aklillu E, Makonnen E, Yimer G, Bertilsson L, Burhenne J, Owen JS (2017) Population Pharmacokinetic Model Linking Plasma and Peripheral Blood Mononuclear Cell Concentrations of Efavirenz and Its Metabolite, 8-Hydroxy-Efavirenz, in HIV Patients. Antimicrob Agents Chemother 61 (8) DOI 10.1128/AAC.00207-17

100 Kitabi EN, Minzi OMS, Mugusi S, Sasi P, Janabi M, Mugusi F, Bertilsson L, Burhenne J, Aklillu E (2018) Long-term efavirenz pharmacokinetics is comparable between Tanzanian HIV and HIV/Tuberculosis patients with the same CYP2B6*6 genotype. Sci Rep 8 (1): 16316 DOI 10.1038/s41598-018-34674-3

101 Mugusi S, Ngaimisi E, Janabi M, Mugusi F, Minzi O, Aris E, Bakari M, Bertilsson L, Burhenne J, Sandstrom E, Aklillu E (2018) Neuropsychiatric manifestations among HIV-1 infected African patients receiving efavirenz-based cART with or without tuberculosis treatment containing rifampicin. Eur J Clin Pharmacol 74 (11): 1405-1415 DOI 10.1007/s00228-018-2499-0
